# 

**Published:** 1860-10

**Authors:** 


### DENTAL REGISTER

### OF THE WEST.

EDITED BY

J. TAFT AND GEO. WATT.

OCTOBER—1860.

---

**MONTHLY:**

**Three Dollars per Annum, in advance.**

---

CINCINNATI:

J. T. TOLAND, PUBLISHER AND PROPRIETOR.

J. TAFT, DENTIST, NO. 56 WEST FOURTH STREET.

